# Retraction Note: Bridge the gap caused by public health crises: medical humanization and communication skills build a psychological bond that satisfies patients

**DOI:** 10.1186/s12939-024-02316-y

**Published:** 2024-11-13

**Authors:** Xiaoou Bu, Yao Wang, Yawen Du, Chuanglu Mu, Wenjun Zhang, Pei Wang

**Affiliations:** 1 Faculty of Education, East China Normal University, No. 3663 North Zhongshan Road, Shanghai, 200062 China; 2 College of Medical Humanities and Management, Wenzhou Medical University, Wenzhou, 325035 China; 3 Key Research Center of Philosophy and Social Sciences of Zhejiang Province, Wenzhou Medical University, Wenzhou, 325035 China; 4 School of Marxism, East China Normal University, Shanghai, 200241 China

**Retraction Note: International Journal for Equity in Health (2024) 23:40**

10.1186/s12939-024-02116-4

The Editors-in-Chief have retracted the article because the authors were not able to provide documentary evidence of ethics approval for the work reported.

Xiaoou Bu, Yawen Du, Chuanglu Mu, Wenjun Zhang, and Pei Wang disagree with this retraction. Yao Wang did not respond to correspondence from the Publisher regarding this retraction.

